# Pathology and Therapeutics

**Published:** 1859-09

**Authors:** Emil Fischer


### PATHOLOGY AND THERAPEUTICS.

BY EMIL FISCHER, M.D.

### I.—*Bronchiectasis Sacciformis.* By PROF. BAMBERGER.

The causes of sacciform dilatation of the bronchia were formerly considered to be only of a mechanical nature; Laennec and his followers believed them to operate in the interior of the bronchial tubes; Corrigan, on the contrary, and in a measure also Rokitansky, looked for them in the surroundings of the tubes. The latter view has been generally accepted in Germany; but it is as unreliable as that of Laennec. Corrigan's opinion is founded upon a misinterpretation of the state in which the pulmonary tissue surrounding the dilatations is found; in most cases it is not solidified, as he assumes, and cannot, therefore, produce a contraction; experience shows, on the contrary, that very often it contains air. Just as erroneous is the view of the same author, that the sac is a kind of compensation for the empty space produced by the obliteration of the air-cells, for in most cases there are extensive portions of the lung impermeable to air, while the dilatations are confined only to small isolated spots; again, cases are not rare in which the portions of the lung in the neighborhood of the dilatation are found to contain more air than those farther removed from it; sometimes even the pulmonary tissue is completely permeable to air. According to Rokitansky, bronchiectasis is caused by bronchitis, complicated with obstruction of the final terminations of the tubes, and collapse of the corresponding parts of the pulmonary tissue; the portions of the bronchia above the obstruction are dilated, partly in order to fill out the vacant space, partly because the air, hindered in its progress, exercises a pressure upon them. The absence of a current of air in the obstructed bronchia speaks against this theory; besides, it does neither explain the origin of bronchiectasis in cases in which the smaller bronchia are not obstructed, and the pulmonary tissue contains air, nor does it account for the sac-like shape of the dilatation. Laennec's theory, that the dilatation is produced by the long-continued stagnation of the mucus, does not explain its sac-like shape; the contents of the tube must accommodate themselves to the original, cylindrical shape, and such is found to be the case on post-mortem examination. With just as little reason the dilatation is ascribed to the pressure of the air on violent coughing.

According to Professor Bamberger, the cause of the dilatation is an atrophic condition of the bronchial tube, brought about by disease limited in its extent. This view is supported by the fact that the membrane of the sac is extraordinarily thin, and that the longitudinal fibres of the wall are found to have disappeared. This atrophy is the consequence of inflammatory action, which is principally confined to the circular layer of muscular fibres, and thus deprives the bronchia of their support against their fluid and gaseous contents. We find, therefore, the bronchiectasis only after chronic catarrh of the bronchial mucous membrane, and after simple or tubercular inflammation of the pulmonary tissue, but not after independent pleuritic exudations; the catarrh is a more important and frequent agency than the inflammation; the latter seems to produce the dilatation only by the simultaneous existence of the former, but not by the induration attending it, although it is not quite impossible that the inflammatory process might extend from the parenchyma of the lungs to the muscular layers of the bronchia. The surrounding pulmonary tissue is in rare cases only quite normal; commonly it does not contain any air, and is discolored by dark pigment; this is always the case, if the dilatations are large and numerous. This condition is produced secondarily by the circumstance that the mucus accumulated in the bronchial tubes, and the compression made by the dilatation, prevent the entrance of the air into the pulmonary cells. In the higher degrees of the affection we often find, therefore, cavities which have no outlet. The process is thus quite analogous to atelectasis. Real induration and callosities are only found when the tissue contains at the same time tubercular deposit; the bronchial dilatation is then often mistaken for a cavity. In these cases there is no causal connection between the changes in the pulmonary parenchyma and those in the wall of the bronchial tube, but both are consequences of a simultaneous inflammation.

Besides these pathologico-anatomical details, Professor Bamberger gives the following statistical items: He observed, in all, eighteen cases of bronchiectasis in which the symptoms were decided; of these, fourteen were male and four were female patients, their age ranging from five and a half to fifty-nine years; most of them being between twenty and thirty years old. Nine of these patients died; in eight cases death was attributable to the disease itself and its consequences; constitution, occupation, and mode of living were indifferent. Of fourteen cases, four suffered previously of pneumonia, and ten of catarrh, during the course of which the symptoms of bronchiectasis were gradually developed. The principal symptoms of the disease are violent coughing fits, with copious expectoration of a peculiarly fetid and purulent sputa, frequent attacks of hæmoptysis, the physical symptoms of the formation of cavities, and the gradual development of marasmus. The fits of coughing are very violent, resembling in younger patients hooping-cough, and are often accompanied by the discharge, in gushes, of many ounces of quite peculiar, not gangrenous, but offensive, and sordid pus, through the mouth and nose, and by vomiting. Dyspnœa is constant, and is owing principally to the stagnation of the pus, and to exacerbations of the catarrh. If there are but few dilatations, and the surrounding tissue contains air, the physical phenomena consist in a râle, with the formation of large bubbles, which remains confined to a limited spot. Such cases can, therefore, not be recognized with certainty during life. In more aggravated cases, symptoms indicating the existence of cavities make their appearance; they resemble those of tubercular cavities, but differ from them partly by their frequent occurrence in the lower and posterior parts of the lungs, and partly and principally by the rapid changes which they undergo; this change of physical signs is owing partly to the peculiar character of the walls, which are not rigid, but yield to pressure, partly to the absence of ciliated epithelium; coughing is thus the only means by which the secretion can be expelled, but it effects a complete evacuation of the sac. The appearance of the patients remains for a long time healthy; later, however, it resembles that of an individual suffering from tuberculosis. Slight dropsy is very common toward the end of the disease; albuminuria was observed only in three cases. The lighter forms of bronchiectasis are not dangerous to life, but more aggravated cases have generally a fatal termination; this is, however, not always the case. Professor Bamberger has observed a cure in a girl, who suffered from an ectasis in the inferior lobe of the left lung; adhesions having formed between the lung and anterior wall of the thorax, it opened externally, and was afterwards obliterated. In three cases the author observed ulceration of the ectasis, with destruction of the pulmonary tissue.

The ectasis generally affects both lungs, especially the inferior lobes; in nearly all cases cylindrical dilatations and catarrh, with thickening of the wall of the bronchial tube, were found to coexist; tubercles were not present in any case.

The treatment consist in a strengthening diet, and the inhalation of the air of mountains and woods; expectoration should be aided by occasionally giving an emetic; in order to correct the fetor, and to reduce the amount of expectorated matter, balsamic medicines and iron should be administered.—(*Oesterreichische Zeitschrift für Praktische Heilkunde,* v. 2, 3, 1859.)

II.—*On the Treatment of Pneumonia.* By DR. BRANDES, of Copenhagen.

The author endeavors to prove, by means of statistics, that the purely expectant treatment of pneumonia has by no means given, at all times and places, those favorable results which its advocates boast of. The indiscriminate use of general bleeding is just as injudicious as the exclusive adoption of the expectant plan.

In every epidemic, it is necessary to determine whether blood-letting is applicable or not to each particular case. Venesection is generally to be avoided in cases where the blood is in an anæmic or dissolute condition, as for instance in drunkards. In the General Hospital, at Copenhagen, the acetate of lead is frequently used in such cases. Professor Christensen praises this remedy very highly, and had ample opportunity of testing its value, as cases of pneumonia in debilitated individuals are of common occurrence in the hospital referred to. Professor Christensen believes the acetate of lead to be one of the most efficacious means in the treatment of pneumonia and prescribes it generally in combination with quinine, one grain of each every two hours. If the cough is very violent, opium is added instead of the quinine. Dr. Bramsen has used acetate of lead, especially in the treatment of pneumonia in very young children, and has been very successful with it. Dr. Brandes, who tried the remedy in doses of half a grain in children of one to eight years of age, has obtained results equally favorable, and extols particularly its calming properties in this disease.—(*Virchow's Archiv*, 1859, xv. 3 and 4.)

III.—*On Carbonate of Lead in Phthisis Pulmonalis.* By PROF. BEAU.

Nature occasionally cures cases of pulmonary tuberculosis; why should we not endeavor to find out its secrets? Professor Beau believes that he has discovered a means of curing the disease; it consists in a pathological antagonism, which, according to him, exists in the effects of lead-poisoning. M. Beau has seen painters, potters, and manufacturers of white-lead, very much enfeebled, but has met with but one of this class of workmen who suffered from phthisis, and in this case the exception was apparent, for the patient had never experienced a single symptom of lead-poisoning.

From this view of a special antagonism between tuberculosis and saturnine cachexia, M. Beau derived the idea of producing artificially an impregnation of the same nature, with the object of making it serve a therapeutical purpose.

It is undoubtedly not the first time that the preparations of lead have been prescribed in pulmonary tuberculosis; but when the acetate of lead was ordered, especially by Professor Fouquier, for the profuse perspiration and colliquative diarrhœa of phthisical patients, this salt was given only as an astringent and palliative; here, on the contrary, the saturnine agent is administered as an alterative, and M. Beau prescribes it with the intention to cure.

Five or six patients in his clinic take *Morton's pills*, a euphemism invented in order to conceal the true nature of the medicine. Morton's pills are composed of balsamic substances; those employed by M. Beau contain only carbonate of lead, in the dose of about one grain and a half for each pill. At first but one pill is taken; the next day he gives two, then three, and thus increases the number every day, until manifest signs of saturnine impregnation are produced, such as jaundice, the blue line at the edges of the gums, and insensibility to pain. He stops as soon as the patient complains of pain in the limbs and of great lassitude.

Among the patients subjected to this treatment, we will mention two women; one of them suffered from an obstinate cough, which provoked vomiting, and prevented her from taking nourishment. In this case the phthisis was characterized by dullness, prolonged expiration, rude respiration, and some crackling; from time to time the cough and muco-purulent expectoration were accompanied by hemoptysis, night-sweats, and by fever occurring in the evening. This patient was easily brought under the influence of the medicine; a dose of three pills sufficed to provoke neuralgic pains, and M. Beau had to reduce the number of pills to two. She suffered for some days even after the cessation of the treatment, but *she did not cough any more.* When she left the hospital the expectoration had stopped completely, and there was no more crackling to be heard; the vomiting had ceased, the patient had an appetite, enjoyed her meals, and was in as good a condition as possible. The other patient was a governess, in whom the disease was still more pronounced. This young person had an enormous cavity below the right clavicle; she expectorated considerably, and the disgust which she felt on account of her sputa, deprived her of all appetite. The treatment with pills of carbonate of lead was commenced on the eighth of April. The impregnation of her system was rapidly effected, and after the number of six pills a day had been reached, it was necessary to diminish it on account of arthralgic pains. But the character of the expectoration was perceptibly modified; the sputa, which was so offensive that it excited a repugnance to its passage and actual vomiting, had lost entirely its disagreeable odor, and was very much reduced in quantity.

Further trials with the treatment recommended by M. Beau are being made at the Hospital Saint André, and a report of them is promised, which we expect to place before our readers in due time.—(*Journal de Médecine de Bordeaux*, June, 1859.)

### IV.—*On Inflammation of the Thoracic Duct.* By Dr. J. Worms.

The occurrence of this disease has been anatomically proved by Andral, Gendrin, and Velpeau. Dr. Worms has had the opportunity of observing it in a man, forty years of age, in the military hospital of Gros-Caillou. The patient was aroused, in the night of the 15th of December, 1858, by a violent pain, seated deeply in the abdomen, and radiating toward both sides; during the following days, this pain subsided somewhat, but violent fever set in. On the fourth day acute pain in the muscles of the forearm supervened, the member becoming red and swollen; then the thighs and the calf of the legs became equally painful, and the evil increased from day to day.

The patient entered the hospital on the 25th of December; the sclerotica was slightly icteric; the lips, tongue, teeth, and skin were dry; the pulse full, hard, and 80 in the minute; the abdomen tympanitic, but not painful. The left arm could not be moved; the anterior and posterior side of the forearm was the seat of considerable tumefaction, and of intense pain. The superficial veins of the whole limb were much distended, and painful on pressure; they presented the peculiarity that it was impossible to make the blood which they contained progress toward the shoulder; while, on the contrary, less resistance was encountered in making it go toward the back of the hand. This circumstance led to the supposition that an obstacle existed to the venous circulation; in examining the whole venous system carefully, no hardness was found except in the left subclavian, which was hard, and rolled underneath the finger. All the other large veins were much distended, and the patient complained of an almost intolerable pain which exactly followed their track. The patient was treated with sulphate of quinia in combination with camphor, in order to combat the general septic condition, and applications of camphorated alcohol were applied to the tumefied arm.

On the twenty-sixth, an aggravation of all the symptoms had taken place; the emaciation had made rapid progress; the patient's look was unsteady; the sclerotica was much more icteric, and the patient was in a state of drowsiness, when not aroused by words. The swelling of the arm was much increased.

During the following days the patient became rapidly worse; the icterus became general, and assumed a shade approaching to green; the intellect was troubled; the evacuations became involuntary, and convulsive movements of the muscles of the lower jaw supervened. The patient died on the thirtieth of December.

*Autopsy.*—All the tissues of the left arm were colored yellow; the aponeurosis was sheathed with an organized fibrinous exudation; all the veins were distended by viscous blood, which was completely discolored, and resembled clear bile. From its passage on the first rib to its junction with the internal jugular, the left subclavian vein was very adherent to the surrounding cellular tissue, and was obliterated by a yellow and hard fibrinous clot.

The whole venous system was distended with uncoagulated blood, and the intestines were much distended by gas. About the cæcum, and in a portion of the ascending colon, deep ulcerations of the isolated follicles existed, without the glands of Peyer being enlarged. The spleen was triple its normal size, and its tissue reduced to a pulpy mass.

The entrance of the thoracic duct into the left subclavian vein was surrounded by an indurated cellular mass; the duct was filled with a large quantity of phlegmonous pus; the receptaculum chyli measured five centimetres in diameter; its walls were colored light yellow, and adhered to the surrounding cellular tissue; the tunics of the whole duct were thickened, and quite opake, the internal coat was softened, deprived of epithelium, and presented small and red ecchymotic spots.

The vertebral column was healthy. Numerous swelled glands surrounded the receptaculum chyli; some of the lymphatics joining it also contained pus. The glands from which these vessels proceeded were white and softened in the part in which the lymphatics originated; the opposite part was hyperæmic and harder. The other viscera, and especially the liver and biliary ducts, presented nothing remarkable.

As there is no reason to assume that the pus was carried into the thoracic duct by one of the branches which unite to form it, the disease consisted evidently in a true lymphangitis of the thoracic duct, and of the receptaculum chyli. The inflammation was propagated to the subclavian vein, and caused there the formation of a clot; this explains the symptoms of stasis of the venous blood in the upper extremity of the left side. In regard to the intense icterus which supervened during the last days, M. Worms looks upon it as a general ecchymosis, produced by the alteration of the blood which was not renewed any more by lymph, and by the stagnation of the circulation caused by a physical obstacle; the icterus had been thus produced without any participation of the liver.—(*Gazette Hebdomadaire*, May 6, 1859.)

V.—*Case of Rupture of the Œsophagus.* By DR. JOSEPH MEYER.

This case occurred, under the author's observation, in Professor Schönlein's Clinic at Berlin. G. S., a shoemaker, of robust frame and intemperate habits, had suffered from time to time since childhood—when he had drank some soap-lye—from difficulty in swallowing solid bodies. A painful weight was then felt at the epigastrium, the morsel requiring great effort to propel it entirely. Of late years, since he had been given to drink, the difficulty had occurred oftener, and did so especially while he was under its influence. On the first of February, a piece of sausage became arrested at the usual spot, and the man made a most violent effort to expel it. The morsel did not return, and a small quantity of bright-red blood was discharged. Great anxiety of countenance and intense pain at the epigastrium were manifested. At the end of about an hour after the commencement of the accident the right side of the face began to swell. A surgeon was called in, and believing the morsel still to be detained, tried to dislodge it, first by emetics, and then by the probang. The patient, obtaining no relief, was brought into the Charité on the fourth of February. He was compelled to maintain the erect position in bed, and derived most ease from bending the body forward. The face was slightly cyanotic, a subcutaneous emphysematous swelling occupying its right side, the neck, and the anterior part of the thorax. The sternum was excepted, and percussion along its course gave out a clear resonance, and was met with abnormally slight resistance. Posteriorly, percussion was dull on the right side, and on the left side it could not be performed on account of the emphysema. Good vesicular respiration was heard all over the chest, with the exception of the lower posterior portion, where it was dull. The respiration was 40; the heart's impulse was feeble, and the pulse was small and soft, and 142. The patient complained of very severe forcing pain at the base of the xyphoid process, which stretched backward to the spine. The diagnosis was rupture of the œsophagus, with some pleuritic effusion, and emphysema consequent upon the rupture. Mustard poultices and ice were repeatedly applied, and the latter given internally, with no relief. Next day, the emphysema had much increased, both in amount and extent, reaching to the edges of the ribs and to the elbows of the upper extremities. The dyspnœa, the cyanotic coloring of the face and hands, and the pain were excessive; and the patient died fifty hours after the commencement of his symptoms.

His body was examined, twenty-two hours after death, by Professor Virchow. At about three inches above the cardia, a gaping, ulcerated surface, an inch and a quarter long and three-eighths of an inch broad, was observed on the anterior wall of the œsophagus. The edges were smooth, and in some spots as sharp as if they had been punched. The mucous coat was destroyed to a greater extent than the muscular. The mucous membrane was nowise thickened, even at the edges, and was easily separable from the muscular coat. The submucous connective tissue exhibited its normal appearance, and the muscular substance was found to be entirely healthy. The coats of the œsophagus in the vicinity of the ruptured parts were in nowise softened. The rest of the œsophagus being somewhat dilated, just above the cardia was a narrowed part, without any distinct cicatricial structure, but having the muscular substance in a hypertrophied condition. In front of the aperture in the œsophagus was a large collection of fluid in the posterior mediastinum, separated from the surrounding organs by a brownish, gangrenous tissue, mingled with the remains of food, and nowhere exhibiting the thickened wall of an abscess. The emphysema had extended from the right side of the mediastinum to the rest of the upper portion of the body. Exudations were found on both pleuræ.

A due consideration of the above case must reject the idea that first presents itself, that the aperture and the large collection of fluid beneath it were the results of old diseased processes; for whether an abscess formed in the substance of the œsophagus or behind this, it would certainly have exhibited at some point a wall due to chronic thickening, and, at the time of the rupture, pus would have been discharged by vomiting. There were none of the morbid characters observed here, described by Albers in his case of rupture following simple ulcer of the œsophagus; and the formation of the collection of fluids may, in great part, have arisen from the employment of the emetics and probang. The difficulty of swallowing complained of by the patient prior to this occurrrence, had doubtless arisen from the narrowing of the cardia, and that such difficulty should be greater when he had been drinking, is explained by the paralytic condition of the muscular coat under the influence of the alcohol,—a state analogous to the dysphagia observed in brain affections, and to the accumulation of food found to take place at the lower end of the œsophagus after the division of the par vagum. We have here, then, a case of rupture of the œsophagus induced by excessive efforts at vomiting, without the parietes being the seat of preliminary ulceration, abscess, or gangrene. So rare are such cases, that the author has only been able to find two recorded with sufficient minuteness for identification; one, the often-quoted case of Boerhaave, and the other communicated by Dryden to the Edinb. Med. Commentaries, vol. iii., 1788. There are other cases of œsophageal rupture on record which are obscure both as to their causes and their symptoms, and some of which were probably dependent upon softening. The course and autopsy of some of these are so imperfectly reported that nothing certain can be said about them. Others, again, are rendered obscure by their complication with accompanying brain affection. The two cases above referred to are quoted at length by the author, and compared with his own.—(*Berlin Med. Zeitung*, 1858, Nos. 39, 40, 41.—*Medical Times and Gazette.*)

VI.—*Observations on the Etiology and Diagnosis of Hepatitis.* By Dr. Bertulus, of Marseilles.

Dr. Bertulus, Physician of the Hôtel-Dieu at Marseilles, has had a practical experience of thirty years, which he gathered, for the greater part, in tropical regions; in accordance with it, he advances the following opinion on the etiology of hepatitis:—

The heat of the atmosphere is not the principal cause of the disease, but miasmatic influences, the use of alcoholic liquors, and stimulating food. Also in tropical climates those regions are comparatively free from the disease where marshy exhalations are not developed; while in northern regions, where intermittent fever is prevalent, diseases of the liver are by no means of rare occurrence. The predisposition to the disease is in this case owing to the periodical swelling of the liver, as it is observed not only in intermittent fever, but also in gastro-enteritis, dysentery, and yellow fever. Yet there are regions free from fever, where diseases of the liver are often observed, and where they are characterized by a peculiar tendency to suppuration, as for instance Andalusia. In these places the principal cause of the disease is the abuse of spirituous liquors and spices; at least, we find that almost all the persons suffering there from affections of the liver are drunkards, or people who eat too much and take their food highly seasoned. In Cadiz, for instance, only sailors, soldiers, and laborers were attacked, all of whom are fond of stimulants; in Malta and Gibraltar the disease did not prevail among the frugal Spaniards, Italians, and Frenchmen, but among the Englishmen, who could not abstain from their accustomed fare, even in a southern climate; in Naples we find the greatest number of patients among the Swiss, who resemble Englishmen in this peculiarity. The same observation is true with respect to the Colonies of Bourbon and Pondichery, and to the first French oriental expedition; in Marseilles, the temperate fishermen who are most exposed to heat, do not fall sick, but soldiers and sailors do. Moreover, it is known that women rarely suffer from diseases of the liver. These views are shared by the physicians of the French navy.

These statements are, of course, not intended to deny that hepatitis may be caused by a general dyscrasia.

The course of hepatitis is often very chronic; in one case communicated by the author, the disease lasted as long as fifteen years.

The diagnosis is often very difficult, particularly in chronic cases; there is always ground for suspicion, if indefinite gastric symptoms persist for a long time.

In the treatment, Dr. Bertulus recommends especially the use of alkaline mineral waters, that of Vichy in particular.—(*Gazette des Hôpitaux,* 17 and 20, 1859.

VII.—*Observations on Fatty, Amyloid Degeneration of the Kidneys.* By Professor Traube.

To the seven cases of fatty, amyloid degeneration of the kidneys, previously published by the author in the *Med. Central Zeitung,* 65, 1858, he adds four other cases, and draws from them conjointly the following conclusions:—

1. Albuminous urine of normal transparency, pale-yellow color, and low specific gravity, with a varied volume, is found in two morbid conditions of the kidneys only, viz.: in shrinking of the organ, which is either the consequence of fatty degeneration or of diffuse, not suppurative nephritis, and in fatty degeneration.

2. If the patient passing such urine exhibits signs of constitutional syphilis, or of a protracted scrofulous disease of the bones, or of chronic tuberculosis of the lungs, the existence of fatty degeneration of the kidneys becomes probable, and so much the more so, if it be ascertained that the disease of the kidneys developed itself subsequently to these diseases.

3. This probability is rendered much greater, if there exists at the same time enlargement of the spleen, or of this organ and the liver, which is neither attributable to intermittent fever nor to an impediment to the venous circulation. Also diarrhœa, without the presence of intestinal ulcers, indicates the presence of the disease.

4. The circumstances mentioned gain importance, if the urine contains not only a large quantity of albumen, but also pus.

5. If there exists at the same time hypertrophy or dilatation of the left ventricle, without valvular disease, we are justified in assuming an extensive degeneration of one or both kidneys.

6. Highly albuminous, heavy, red urine, the color of which depends upon an abnormal quantity of coloring matter, is observed in fatty degeneration of the kidneys, if in the course of the malady an acute febrile disease is developed, or a structural change in the respiratory or circulatory apparatus takes place which is capable of reducing the tension of the aortic system to a considerable degree; perhaps this condition of the urine may also occur when the epithelium becomes extensively implicated in the fatty degeneration, before the strength of the patient is much exhausted.—(*Deutsche Klinik*, i. 7 and 8, 1859; *Schmidt's Jahrbücher*, May, 1859.)

VIII.—*On the Treatment of Prurigo.* By PROF. V. BARENSPRUNG.

Professor Bärensprung does not agree with Mr. Hunt in the high estimate he forms of the utility of arsenic in obstinate prurigo. He has derived just as little service from the use of Fowler's solution as from the internal use of tar, antimonials, or sulphurets; and he feels much more disposed to agree with Rayer that, independently of certain modifications which the condition of the constitution may require in certain cases, external means are those which should be exclusively relied upon. But the choice of topical applications is not a matter of indifference, for they are quite as capable of doing harm as good. The employment of strong stimuli should be avoided, and even the soap frictions recommended by Hebra and others have always done harm in the author's hands, the free alkali they contain giving rise to increased heat and itching, and in sensible skins inducing eczema. Yet more objectionable are strong alkaline washes. Local anæsthetical applications, as chloroform, and other articles, are useless. A temporary relief is almost always obtained by cupping, which diminishes the afflux of blood that takes place during the paroxysms. Cold baths and applications are usually employed for the momentary relief which they afford. The relaxing influence of tepid or bran baths, and the transpiration induced by a vapor bath, calm the state of excitement which spreads from the cutaneous nerves to the entire nervous system. As a no less useful calming application, inunction of the surface with mild fats, or with bacon-fat, may be resorted to.

The above means cannot be regarded as curative agents. As such, we may mention sulphur, tar, and baths of corrosive sublimate. By means of sulphur, mild cases of prurigo may often be cured; yet we must take care and not be deceived with regard to its effects in an affection so liable to natural periods of remission. It may be used in the form of baths or of ointments; and the author refers to the prompt and lasting relief derived by an aged person in an obstinate prurigo, from the use of the *unguent sulphur. anglicum.* From ointments made of the different varieties of tar he has also derived undoubted benefit, preferring *oleum cadinum* (one part to two of fat) as the least disagreeable of remedies. Cases which have resisted the above remedies have yielded to tepid sublimate baths; two or three of these, each containing two drachms of the sublimate, rapidly dissipating a long-standing affection, the mercury seeming to exert only a local influence. The bath should not be taken in a metallic, but a wooden vessel.—(*Annale der Charité Berlin,* viii. 3.—*Medical Times and Gazette,* June, 1859.)

### IX.—*Observations on Megrim.* By Prof. Piorry.

In a clinical lecture, published lately in the *Gazette des Hôpitaux,* is contained some interesting remarks by Professor Piorry on the theory and treatment of the nervous affection commonly known as "megrim," and to which the learned nosologist has given the name of "irisalgia."

Persons subject to "megrim," says M. Piorry, know its approach by the appearance of a cloud covering the centre of the field of vision, such as may be produced on the retina of a healthy person by looking at any very brilliant object. The circumference of the cloud is finished off by ten or twelve teeth placed in a zigzag manner, and the whole extent of the central cloud is of a bluish tinge, resembling the electric light or that of the stars. This image has an incessant oscillating motion, which is followed sooner or later by vomiting, and in some cases by a tingling sensation in the median and ulnar nerves, and persons completely blind from amaurosis have, during the attack, observed glimpses of light. This luminous figure or spectre follows the movements of the eye; it is seen more vividly while the lids are closed, and when they are open, objects are seen only round its circumference; thus, on looking at a person's face, the middle portion is not seen. Where do these changes take place? Is it in the retina? That is doubtful, although the optic nerve is the real origin of the phenomena of vision. M. Piorry inclines rather to place the true seat of the affection in the iris, on account of the vomiting which supervenes on it, because surgeons have long remarked that if, in the operation for cataract, the iris be touched, vomiting comes on almost immediately. This is explained by the anastomosis of the third and fifth pair with some filaments of the pneumogastric.

Here is, then, a primary pathological fact attested by a number of intelligent persons who have experienced it themselves, viz., MM. Sabarraque, Foucault, Jules, Pelletan, Subanski, etc. This fact of itself, indisputable as it is, is sufficient, according to M. Piorry, to establish this theory.

Meanwhile, how must this "megrim" be treated? M. Piorry says it may be arrested in the outset by functional stimulants—that is to say, suitable articles of diet; for instance, two ounces and a half of Bordeaux wine, with a sufficient quantity of bisçuit. In cases in which the megrim relapsed with great intensity, M. Piorry administered the following draught, from which he has experienced unhoped-for success, and which appears to him to be indicated wherever the nervous system is involved: ℞.—Quinine, gr. xxx; alcohol, or tincture of canella, enough to dissolve it; water, to increase the amount without precipitation; syrups, half an ounce.

The object which M. Piorry has in view in prescribing this draught is to produce in the nervous system sudden vibrations sufficient to neutralize those already existing. In a word, it acts by substitution, and this much is certain, that the quinine draught cures even those cases of neuralgia which do not assume the periodic form.—(*Journal de Médecine et Chirurgie.—Dublin Med. Press*, June, 1859.)

### X.—*On the Treatment of Ischias.* By PROF. TROUSSEAU.

After a lengthy discussion on the two forms of ischias, the idiopathic and the symptomatic, which offers, however, nothing new, Professor Trousseau speaks of the treatment. It is the same in both forms, and consists first in the administration of oil of turpentine, a remedy the efficacy of which, in the disease in question, is well known, and which, if it is borne by the patient, offers very favorable results. Professor Trousseau prescribes it in the dose of about two to three drachms in the day, and directs it to be taken in capsules, particularly those of Mesery, which contain a little over fifteen grains, and can be filled by the patient himself. In very violent cases, he combines with it the extract of belladonna.

The second method consists in the application of an issue in the region of the sciatic notch. During the day, Professor Trousseau keeps it open by means of issue peas, while for the night he fills it with pills consisting of Extr. Belladonnæ, Extr. Opii, āā gr. j; Pulv. Guaiaci, gr. iv; and Gummi Tragac. q. s.; these pills are very hard, but dissolve slowly in the issue. He uses them only during the night, in order to avoid their general narcotic effect. After the pain has disappeared, he keeps the issue open for one to two weeks by peas.—(*Clinique Européenne*, 3, 1859.)

### XI.—*Small Doses of Ipecacuanha in Obstinate Malarious Intermittent.* By DR. C. SAUREL.

In a case of obstinate intermittent, occurring in a young man, twenty years old, the author, after employing quinine, arsenic, apiol, etc. in vain, prescribed a powder of about four grains and a half of ipecacuanha, to be taken every morning, and cured the patient by this treatment within a week. The medicine produced neither nausea, vomiting, nor any other of its physiological effects.

Dr. F. Roux, of Cette, remarks, in a letter addressed to the editor of the *Revue Thérap. du Midi*, (March,) that the favorable action of ipecacuanha in cases of this kind has been known to homœopaths for a long time.—(*Rev. Thérap. du Midi*, 13, p. 108, February, 1859.)

### XII.—*Substitute for Cod-liver Oil.* By Dr. Hobbs.

The oil of the dugong has been lately proposed as a substitute for cod-liver oil, by Australian physicians. Dr. Hobbs, Medical Health Officer of Moreton Bay, Australia, has again called attention to its curative properties; and if this oil should prove as efficacious in the hands of others as he believes it has been in his own, there will be great reason for congratulation. The dugong is very abundant in the Australian waters and in the Indian seas, and might be obtained in large quantities at a moderate cost. It has the advantage of being a pure, sweet, and palatable oil, which may be used in cooking, and is peculiarly digestible. On the other hand, it does not contain iodine. Those who look upon the iodine of cod-liver oil as an active and important agent in the production of its peculiar effects, will probably be disinclined to accept this report in all its details; it strengthens the position of those physicians who believe that cod-liver oil is mainly valuable as supplying, in a digestible form, carbonaceous material, and thus sustaining enfeebled vitality. Dr. Hobbs prescribes dugong oil in those cases in which cod-liver oil is usually prescribed, and his success has been very encouraging. He believes that it is particularly well suited to the form of disease known as "dyspeptic phthisis." The statements of Dr. Hobbs are well worthy the attention of the profession; and if it be found that the dugong oil can be substituted advantageously for cod-liver oil, the gain will be great to the patient, who will thus be enabled to procure an agreeable and palatable oil at a much less cost than that of the more nauseous and unpleasant medicament now in such high esteem.—(*London Lancet*, June, 1859.)

### XIII.—*On the Poisonous Properties of Oil of Elemi.* By Dr. E. Mannkopf.

The experiments of Mitscherlich, (Pr. Ver. Ztg., 45, 1843, and 19 and 20, 1848,) J. Simon, (De ol. æth. junip. vi, Diss. inaug. Berol., 1844,) and Goedecke, (De ol. æth. Cubeb. exper. nonnull., Diss. inaug. Berol., 1850,) with some of the group of ethereal oils, containing 5 C. and 8 H., induced the author to investigate the properties of the ethereal oil of elemi, which shows a similar composition. The results of his experiments are the following:—

The resin of elemi contains about six per cent. of an ethereal oil, which is transparent, nearly colorless, of not an unpleasant smell, and of a somewhat acrid, bitter taste. Sp. gr. 0·852, boiling point between 166 and 174° C.; it burns with a shooting, bright flame, is insoluble in water, easily dissolved in alcohol and ether, is colored red by $SO_3$, detonates with fuming nitric acid in the cold, leaving a resinous, tenacious mass of not unpleasant smell. The same takes place with the nitric acid of the pharmacopœia on heating. Its formula, according to Stenhouse and Deville, is $C_5 H_8$.

The experiments were made on rabbits and frogs. In the rabbits the oil was injected into the stomach through an elastic catheter; in the frogs it was introduced partly in the same way, partly it was injected underneath the skin of the back, partly applied locally to different parts. The author draws the following conclusions from his experiments:—

Introduced into the intestinal canal, the oil produces in the stomach hemorrhagic erosions and various functional disorders; in the intestines, increased peristaltic motion and pain, which are followed by paralysis and anæsthesia. In a similar manner the oil acts on any part when directly applied. After being absorbed, it paralyzes the nerves of sensation and the par vagum. It produces, accordingly—1. An increase in the rapidity and force of the movements of the heart; from this results an increased diuresis, which is accompanied by inflammation of the kidney, and, perhaps, also of the bladder, this inflammation being caused by specific irritation, and assuming different degrees of intensity; the augmented diuresis causes also an increase of thirst. 2. A restraint and gradual cessation of respiration, which produces atelectasis, with œdema of the lungs, and a decrease of temperature, an additional cause of which, however, is defective nutrition. The second point, in connection with the paralysis of the heart, which finally sets in, causes death; may be that cessation of the cerebral functions assists in bringing about this result.

With reference to therapeutics, the oil may become useful by increasing the contraction of the heart, by augmenting the diuresis, and by producing anæsthesia of the nerves of sensation. The author believes that the oil is not likely to be called into use on account of the first of these properties, as in most cases milder excitants would be preferable. With regard to the second property, the oil of elemi might be usefully employed in all cases in which oil of turpentine is indicated, its pleasant taste making it preferable to the latter. Also, on account of the third property, it could be made a useful substitute for oil of turpentine, for instance, in cases of neuralgia. Whether the therapeutic application of the oil of elemi is attended with any danger, on account of the inflammation of the kidneys, which may possibly arise, must be ascertained by experiments on the living subject.—(*Virchow's Archiv*, 15, p. 192, 1859.)

### XIV.—*Selenite a Substitute for Quinine.* By MR. S. CLARK.

Mr. Clark, Civil Assist. Surg. Bengal Medical Service, recommends, in a letter to the editor of the *Medical Times and Gazette*, selenite as a substitute for quinine. In the month of September, 1858, while investigating the use of some preparations of arsenic, much in vogue among the natives in India as remedies in fever, he found that the one most valued as a febrifuge, a fine impalpable white powder, under the name of hurtal, (the common native name of arsenic,) was not arsenic, but some other substance insoluble in any of the strongest acids at his disposal. He, however, commenced its use as a febrifuge, in small doses at first, and the results have been so satisfactory that he considered it his duty to bring so cheap and useful a remedy to the notice of the profession.

On further inquiry, he found the substance was the product of a mineral in a calcined form, procurable in most bazaars; and subsequently pronounced by Dr. Macnamara, Chemical Examiner to the Government, to be selenite.

The natives calcine the mineral in combination with the fresh pulp of the India aloe. About equal parts of each are put into two small earthen pots luted together, and placed on a slow fire. This rough crucible is allowed to cool gradually as the fire burns down, the whole process taking about twelve hours.

Mr. Clark prescribes the medicine in five to ten-grain doses for adults, and one to three grains for children, every four hours, in intermittent fever, and in other cases requiring tonics, and finds it an excellent substitute for quinine, both alone and in combination with other medicines. He has been in the habit of using it almost daily for some eight months.—(*Med. Times and Gazette*, June 11, 1859.)

XV.—*The Acid Nitrate of Silver.* By M. Crocq.

M. Crocq lately read a paper before the Medical Society of Brussels, wherein he sets forth the advantages of a caustic solution hitherto not much employed, namely, the acid nitrate of silver. The author states that it should be used when the surface acted upon is to be more or less deeply modified, without an intention of destroying much thickness of tissue, in fact, in those cases where the solid nitrate of silver or the acid nitrate of mercury are generally used. The acid nitrate of silver is, however, superior to the simple nitrate, as it penetrates much better into interstices, and as its action may at will be made superficial or deep, (the difference depending on the longer or shorter contact.) It is also preferable to the acid nitrate of mercury, because it produces no tonic effects, and never gives rise to alarming symptoms, however extensive the surface may be with which it is brought in contact. Nor can it excite salivation. Its action can, moreover, be at once stopped when the vagina, the mouth, or the eye, are operated upon, as an injection of a solution of common salt will immediately render it inert. Chancres, simple or sloughing ulcers, hospital gangrene, lupus, epithelial cancer, etc. can be treated with this caustic solution. It may be prepared either from the simple nitrate or from metallic silver. To obtain it from the lunar salt, it will be sufficient to add eight times by weight of nitric acid, at thirty-three degrees, to the nitrate of silver, and expose to heat in a stopped bottle. With metallic silver, ten times the weight of nitric acid, at thirty-five degrees, should be poured on the metal, and a gentle heat be used.—(*Bull. de Thér.*, 46, p. 140, February, 1859.)

